# Bilateral Inverted V-shaped High Tibial Osteotomy: A Case Report

**DOI:** 10.7759/cureus.54558

**Published:** 2024-02-20

**Authors:** Aboubacar Lawan Abdou, Taha El Aissaoui, Adnane Lachkar, Najib Abdeljaouad, Hicham Yacoubi

**Affiliations:** 1 Department of Orthopedics and Traumatology, Mohammed VI University Hospital, Faculty of Medicine and Pharmacy, Mohammed First University, Oujda, MAR

**Keywords:** hto, tibia, inverted v, osteotomy, high, bilateral, severe deformity, genu varum, knee

## Abstract

The article describes the case of a young patient with bilateral genu varum deformity, limiting her mobility. The therapeutic decision was a staged reverse V-shaped tibial osteotomy on both knees at a six-month interval. The surgery faced infectious complications on the left side, requiring additional treatment. Despite this, the patient achieved successful correction, with wound healing and bone consolidation. Preoperative planning was crucial, determining specific correction angles for each knee. The reverse V-shaped osteotomy demonstrated satisfactory functional outcomes compared to other techniques. The conclusion emphasizes the effectiveness of reverse V-shaped high tibial osteotomy (HTO) in addressing varus tibial deformities, providing an alternative before considering total knee arthroplasty. Multicenter studies and long-term evaluations are recommended to refine this surgical procedure.

## Introduction

The knee joint is subject to significant loads, bearing multiple times the body weight during daily life activities. It is a highly complex joint, and its stability primarily relies on the interaction of ligamentous, meniscal, muscular, and tendinous structures, cartilage, and bone [1]. The absence of automatic progression to osteoarthritis in this joint is attributed to the perfect distribution of loads achieved through the interaction of these elements and proper alignment. Osteotomies play a crucial role in balancing the mechanical load between the two tibiofemoral compartments by correcting abnormal joint alignment [1,2].

We present the case of a young patient who underwent bilateral high tibial osteotomy (HTO) within our department.

## Case presentation

We report the case of a 21-one-year-old female student, apparently without significant medical history, presenting with mechanical pain in both knees and a progressively developing bilateral varus deformity, increasingly restricting the walking perimeter. The patient reported that the deformity had slowly manifested since the age of twelve. At that time, she had received realignment orthoses, but without clinical improvement.

**Figure 1:**
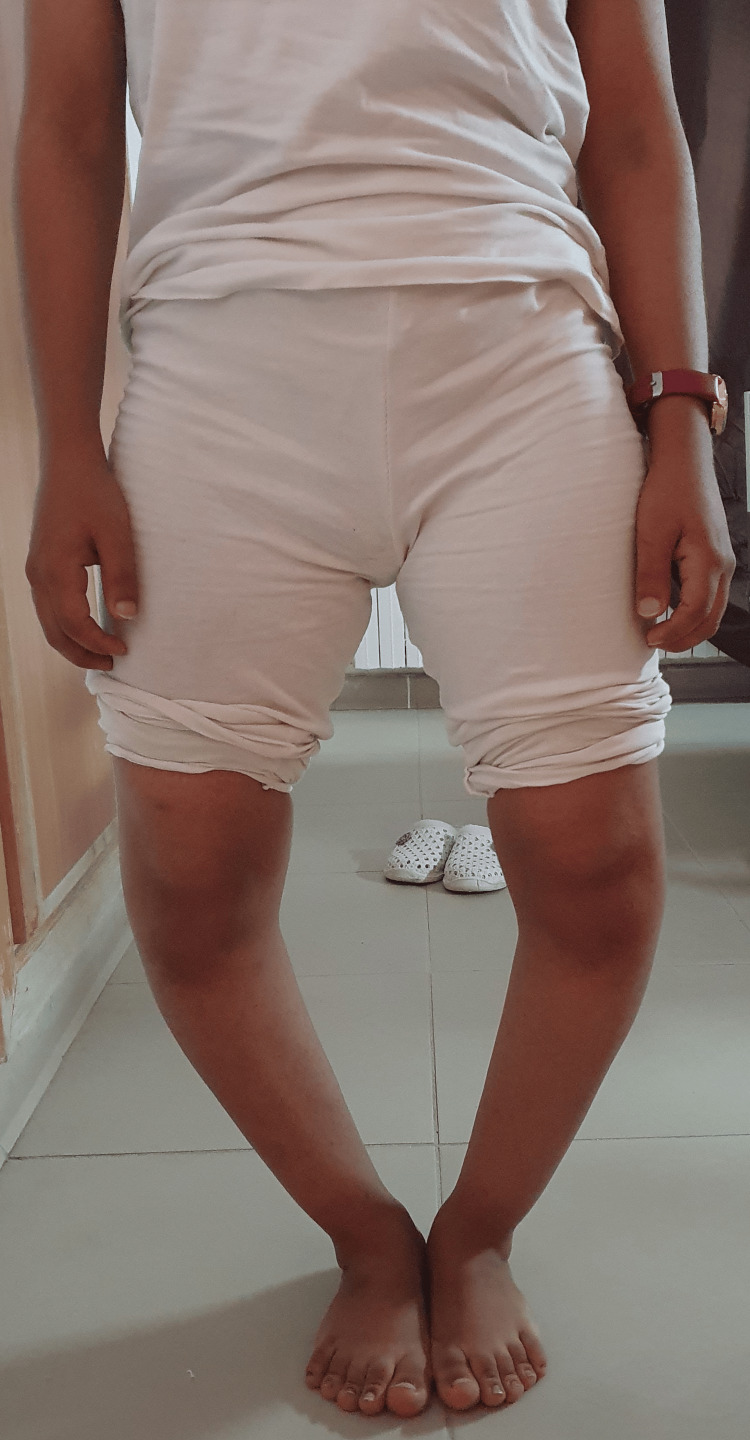
Clinical appearance revealing a pronounced bilateral genu varum deformity.

**Figure 2:**
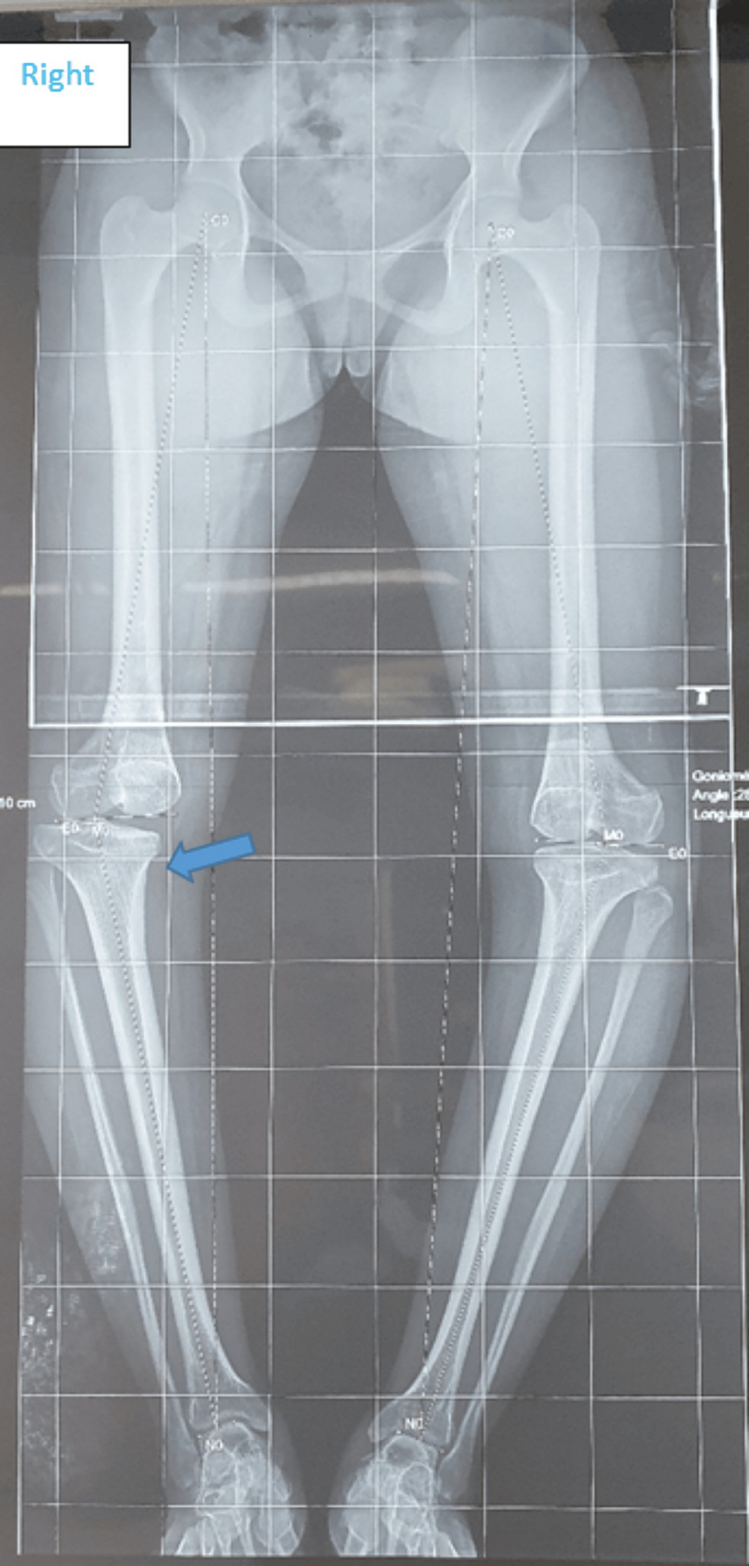
Preoperative pangonogram revealing the location of the deformity. The blue arrow indicates the tibial location of the deformation.

Upon general examination, the patient was in good overall health without apparent dysmorphic features. Walking was possible but accompanied by a noticeable limp. Standing revealed a bilateral genu varum morphotype with a width of 10 fingerbreadths (19.5 cm), persisting even in the supine position (Figure 1). Joint ranges of motion in both knees were preserved. Palpation of the joint space did not elicit pain. Both hips were free and painless, and the spinal examination revealed no particularities. The patient underwent a long-leg standing X-ray (Figure 2).

**Figure 3:**
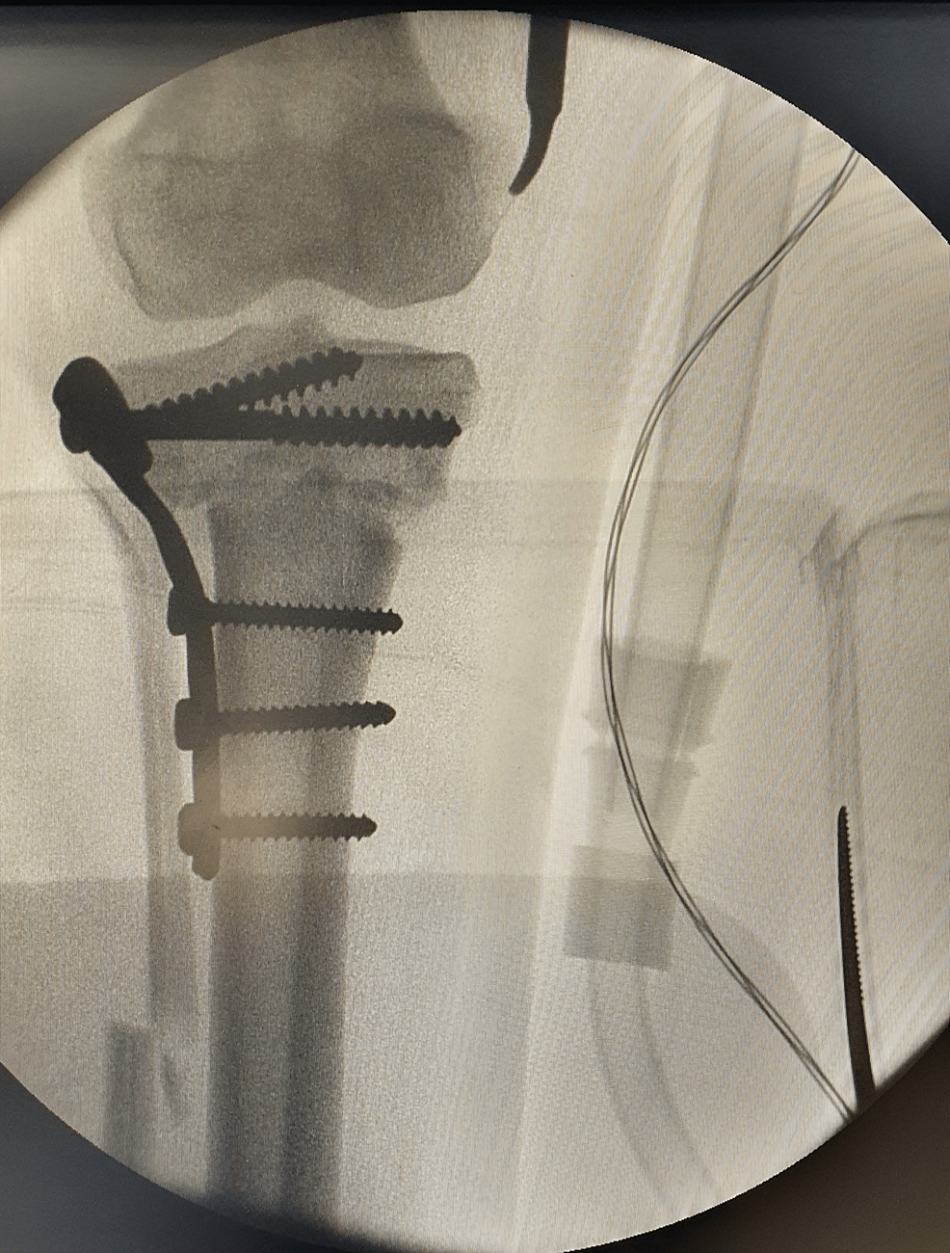
Intraoperative fluoroscopic view of the right knee demonstrating correction and stabilization with a plate.

**Figure 4:**
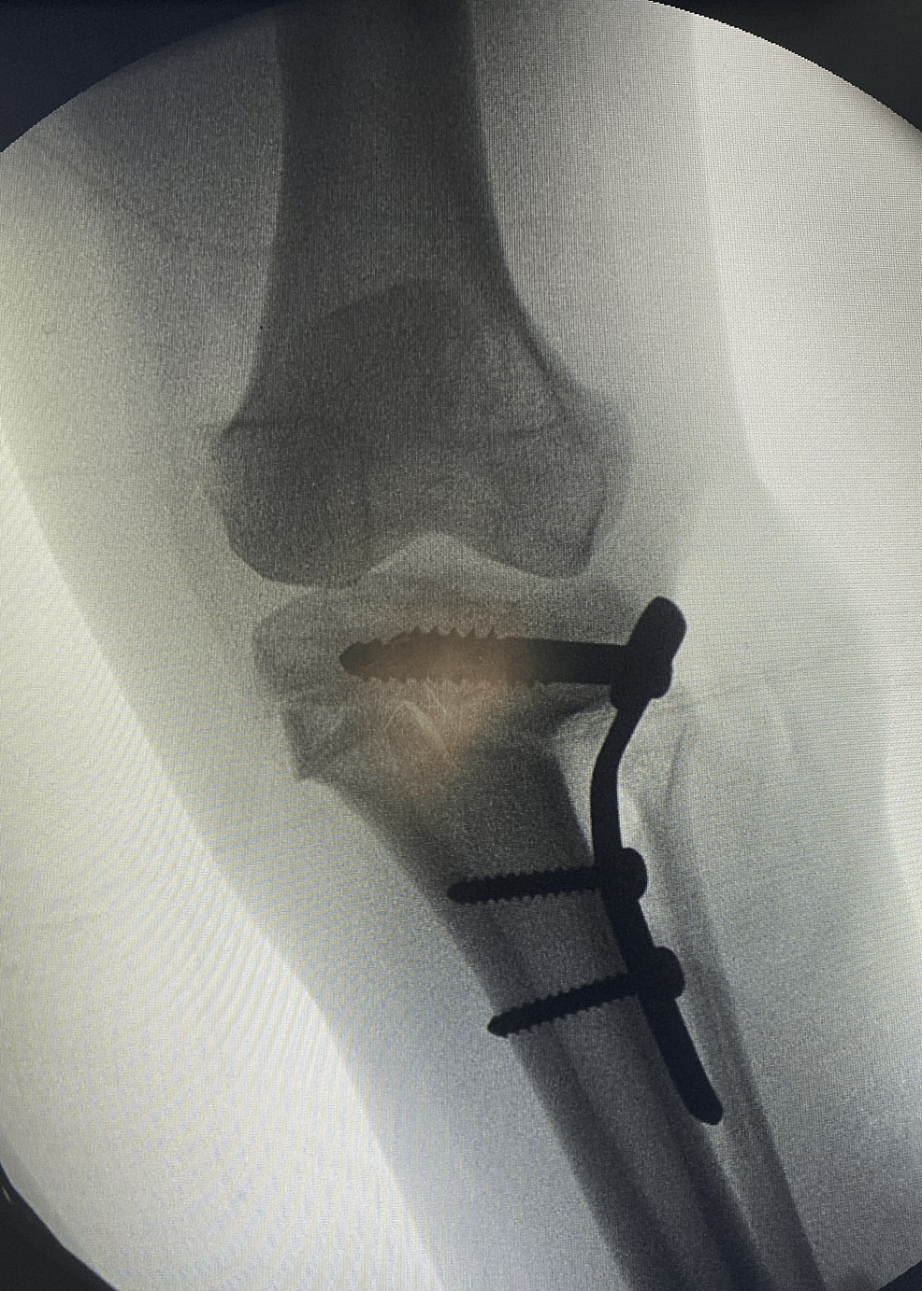
Intraoperative fluoroscopic view of the left knee demonstrating correction and stabilization with a plate.

The therapeutic decision was to perform a two-stage osteotomy on both knees with a six-month interval between each procedure. We conducted a "V-shaped" osteotomy on the right knee, followed by the left knee (Figures 3, 4). The postoperative course was uneventful for the right knee, while for the left knee, complications arose, including purulent drainage on day 19 accompanied by a fever reaching 39°C, suggestive of a postoperative infection. Management involved surgical debridement, plate replacement, and cytobacteriological sampling. Analysis revealed *Staphylococcus aureus* sensitive to ciprofloxacin and ceftriaxone.

**Figure 5:**
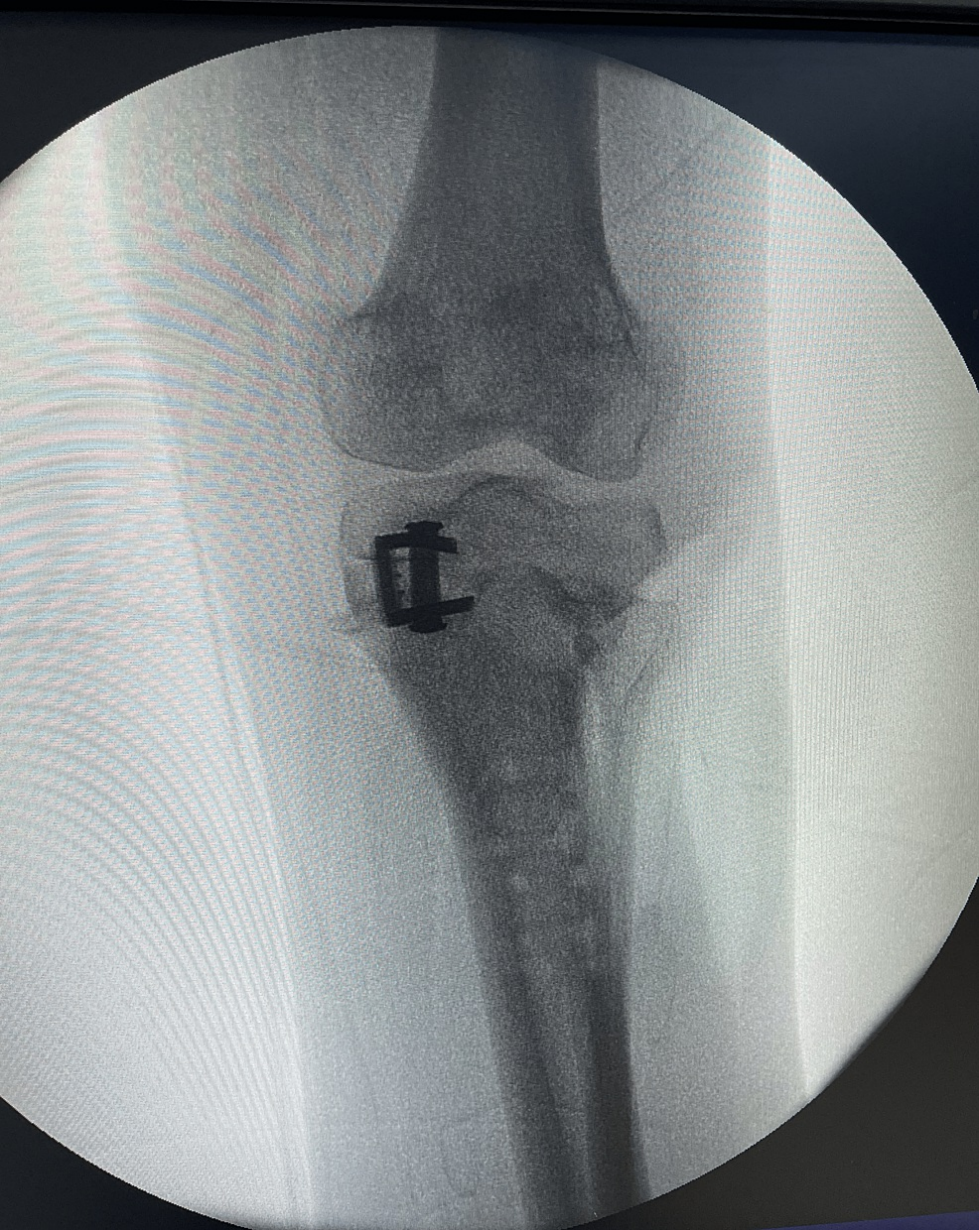
Fluoroscopic view showing post-plate removal and staples placement.

Two months later, arthroscopic lavage was performed, followed by another procedure a month later, involving further debridement and replacement of the plate with two staples (Figure 5). These interventions led to drying of the operative site and bone consolidation in less than two months.

**Figure 6:**
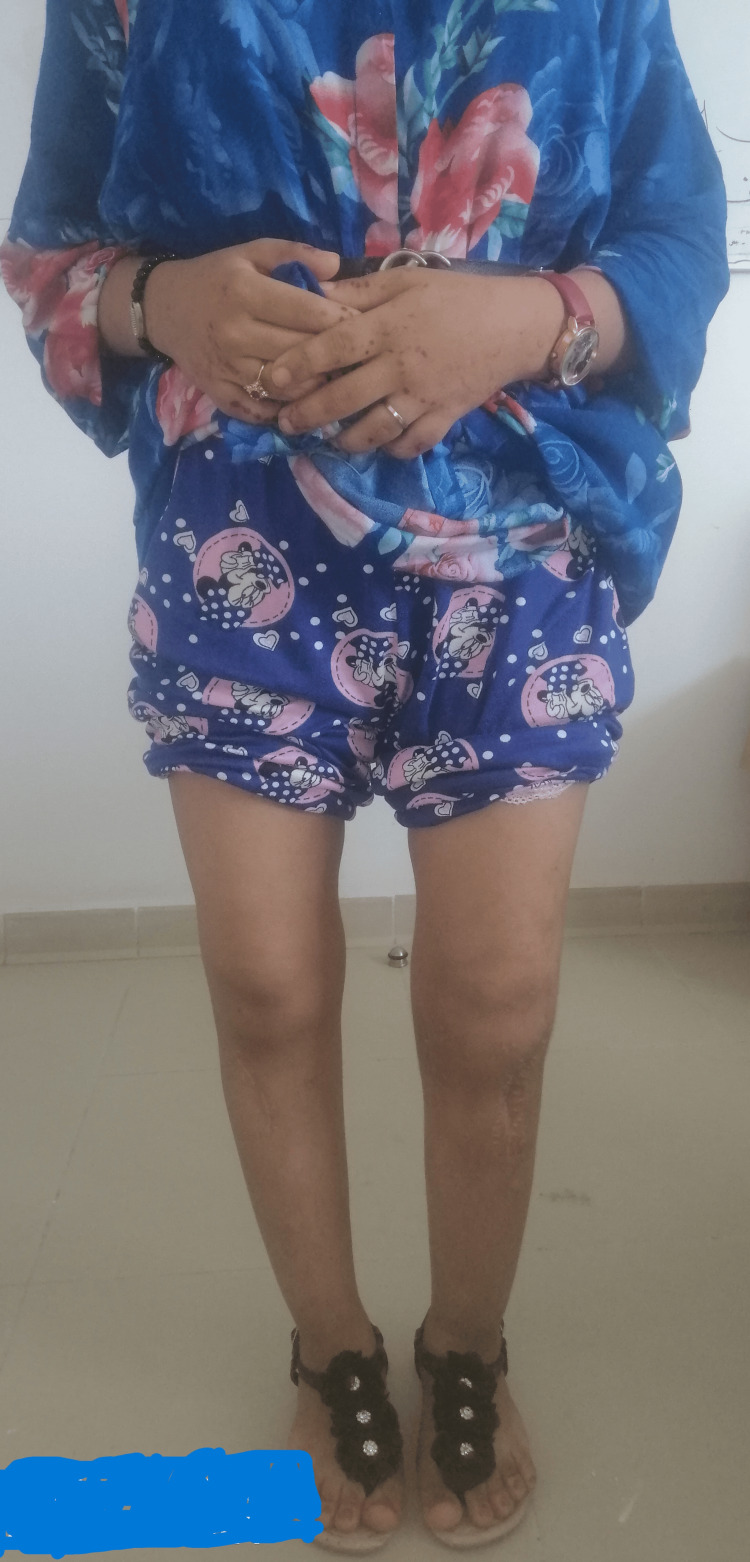
Clinical appearance of correction at 12 months from the first surgery.

Currently, the patient walks without pain or mechanical aid, and she is satisfied, resuming her daily activities seven months after the osteotomy on the second side (Figure 6).

**Figure 7:**
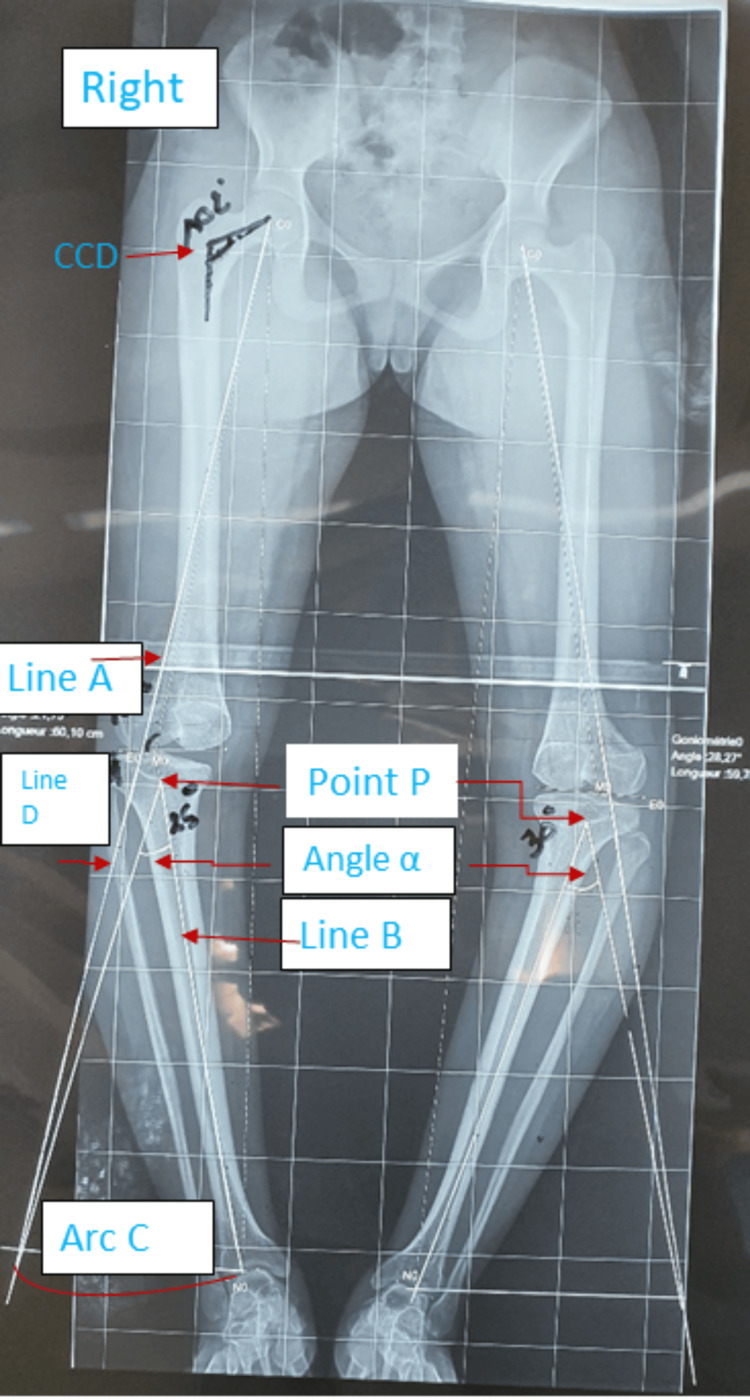
Preoperative planning detailing correction angles, 25° on the right and 30° on the left. Line A, line B, line D, and arc C are drawn to calculate angle α. Determination of the point P at 3 centimeters from the joint surface. CCD: Hip cervico-diaphyseal angle.

Preoperative planning is a crucial step in the management process. It is conducted based on a pangonogram, allowing for the determination of the required correction. We identified a correction angle of 25° for the right knee and 30° for the left knee (Figure 7). The decision to start with the right side was influenced by the intensity of pain reported by the patient. This personalized approach aims to maximize the benefits of the osteotomy while taking into consideration the specific symptoms experienced by the patient.

## Discussion

The operative technique is as follows. The initial step involves performing a fibular osteotomy at the junction of the upper and lower two-thirds through a small lateral incision. Following this, a reverse V-shaped tibial osteotomy is carried out using an antero-external approach, guided by preoperative data (Figure 7). The entire right lower limb radiograph is employed to outline the lines for the inverted V-shaped osteotomy. The apex point (Point P) is positioned at the center of the tibial condyle, approximately 3 cm from the joint surface line, with an apex angle of around 110°. To ascertain the angle for lateral hemi-wedge resection, a long line (Line A) is drawn from the center of the femoral head through the 65% or 62.5% point on the lateral tibial plateau. Subsequently, another line (Line B) is drawn from the apex point (Point P) to the center of the talar dome, and the length of Line B is measured. An arc (Arc C), with the apex point (Point P) as its center and Line B as its radius, is drawn intersecting Line A. Another line (Line D) is drawn from the apex point (Point P) to the intersection point of Line A and Arc C. The angle (α) formed between Lines B and D represents the lateral hemi-wedge resection angle, which is identical to the correction angle of the lower limb alignment. Using the angle (α), a dashed line is drawn to represent the lateral hemi-wedge resection line [3].

As with any knee deformity, it is crucial to first determine the origin of the malalignment: femoral, tibial, or mixed [3]. In varus knee deformities, only 28% of patients exhibit an exclusive tibial localization and are potential candidates for HTO [4]. This involves a meticulous analysis, using a pangonogram, of certain angles such as medial proximal tibial angle (MPTA), mechanical lateral distal femoral angle (mLDFA), the mechanical axis of the femur, joint-line convergence angle (JLCA) [2,5,6].

Once the tibial origin is confirmed, the next step is to calculate the correction angle. A first line is drawn from the center of the femoral head to the center of the talus. The second line passes through the femoral head and the Fujizawa point, located between 65 and 70% (from internal to external) of the proximal tibial articular surface for Kellgren-Lawrence stages 2 and 3, or at 62.5% if stages 1 or 2 [3].

Aoki et al. argue that there is a lower risk of delayed consolidation and loss of radiological correction with the reverse V-shaped osteotomy [7]. This technique is considered reliable, with functionally satisfactory results (according to the Japanese Orthopaedic Association functional score) at 83%, compared to 63% with a closing wedge osteotomy. It should be noted that, in this comparative study, the preoperative status of both groups was similar [7]. Maintaining correction, even after the removal of the osteosynthesis material, is considered by several authors as a guarantee of a good long-term result with a correction angle [8,9].

Various complications have been reported in the literature. In order of frequency, these include fractures of the cortical hinge, implant pain, loss of correction, fibular nerve injuries, surgical site infections, and pseudarthrosis or delayed consolidation [10,11]. Infection was the only complication observed in our patient. The reverse V-shaped HTO is a reliable method to delay or even halt joint destruction in often young patients with significant tibial varus deformity. This allows postponing the radical solution of arthroplastic replacement without excluding it altogether [12,13].

## Conclusions

High tibial osteotomy (HTO) serves as a solution to improve knee function and delay or even avoid the potential need for arthroplasty. The practitioner should always consider the possibility of subsequent total knee replacement because joint deterioration, though significantly slowed, may eventually progress to the ultimate stage of osteoarthritis. In conclusion, multicenter case series studies and long-term evaluations are imperative to refine this surgical procedure and achieve even better functional outcomes.
